# Sustainable under nutrition reduction program and dietary diversity among children’s aged 6–23 months, Northwest Ethiopia: Comparative cross-sectional study

**DOI:** 10.1186/s12939-019-1120-1

**Published:** 2020-01-28

**Authors:** Tigist Worku, Kedir Abdela Gonete, Esmael Ali Muhammad, Asmamaw Atnafu

**Affiliations:** 1 Bahirdar Zuria Woreda, Ethiopia; 2 Department of Human Nutrition, Institute of Public Health, University of Gondar, College of Medicine and Health Sciences, P.O. Box 196, Gondar, Ethiopia; 3 Department of Health Systems and Policy, Institute of Public Health, College of Medicine and Health Sciences, University of Gondar, Gondar, Ethiopia

**Keywords:** SURE program, Dietary diversity. Children aged 6–23 months, West Gojjam zone

## Abstract

**Introduction:**

Adequate dietary diversity is vital for the survival, growth and development of infants and children. Inadequate dietary diversity is the major cause of micronutrient deficiency in Sub-saharan Africa, including Ethiopia, where only less than one-fourth of the children aged 6–23 months obtain adequate diversified diet. Thus country implemented a strategy known as the Sustainable Undernutrtion Reduction (SUR) programs to alleviate the problem. However, empirical evidences are scarce on the impact of the program on children aged 6–23 months. Therefore, this study aimed to compare the level of dietary diversity among children aged 6–23 months in districts covered and not covered by SURE program in West Gojjam zone.

**Methods:**

A community based comparative cross-sectional study was conducted in three districts of West Gojjam zone, Ethiopia, from February 29 to April 20, 2019. A total of 832 mother and child pairs were selected by the simple random sampling technique. A pretested and structured interviewer-administered questionnaire was used to collect data. A binary logistic regression model was fitted to identify factors associated with dietary diversity. Crude odds and adjusted odds ratios with 95% confidence intervals (CI) were calculated to assess the strength of associations and significance of the identified factors for dietary diversity score.

**Result:**

The overall proportion of adequate dietary diversity among children aged 6–23 months was 29.9% (95% CI: 27.0–33.0), whereas in SURE covered and uncovered districts it was 33.4% (95%CI: 29.0–38.and 26.4%(95% CI: 22.0, 31.0), respectively. ANC (Antenatal care) (AOR = 1.7; 95% CI: 1.16, 2.55) and postnatal care services (AOR = 2.1; 95% CI: 1.38, 3.28), participating in food preparation programs (AOR = 1.9; 95% CI: 1.19, 2.96), GMP (AOR = 2.74,95%CI:1.80, 4.18), vitamin A supplementation (AOR = 2.10,95%CI:1.22, 3.61) and household visits by health extension workers (AOR = 2.0; 95% CI: 1.25, 3.21) were significantly associated with dietary diversity.

**Conclusion:**

The proportion of adequate dietary diversity was higher among children in the program than those out of the program. ANC visits, PNC follow-ups, women’s participating in food preparation programs and household visits by health extension workers were significantly associated with dietary diversity. Therefore, and strengthening and scaling up the program to non covered districts and providing health and nutrition counseling on Infant and Young Child Feeding (IYCF) during ANC and PNC services are recommended for achieving the recommended dietary diversity.

## Introduction

Dietary diversity (DD) is the number of food groups consumed over a reference period and used as a proxy indicator of dietary quality and nutrient adequacy [1]. According to the World Health Organization (WHO) 2011, children aged 6–23 months should consume at least four of the seven food groups, like; grains, roots, and tubers, legumes and nuts, dairy products, flesh foods (meats/fish/poultry), eggs, vitamin A-rich fruits and vegetables, and other fruits and vegetable [2].

Globally, nearly half (45%) of the deaths among under- five children occurred due to poor nutrition [3], and the magnitude of under-nutrition in 2016 indicated that 155 and 55 million children were estimated to be stunted and wasted respectively. The burden was not evenly distributed because the bulk occurred in South Asia and Sub-saharan Africa [4]. Sub-saharan Africa, especially East and West Africa, accounted for the highest load (57.7%) of child malnutrition [5]. According to the Ethiopian Demographic and Health Survey (EDHS) 2016 report, 38, 10, and 24% of the children were stunted, wasted, and underweight, respectively. In addition, only 14% of the children aged 6–23 months received the minimum dietary diversity [6].

Insufficient quantity and quality of diversified diets are among the cause of under-nutrition in developing countries [2]. The diets of Ethiopian children predominantly contain on starchy staples and often include little or no animal products and little fresh fruits and vegetables [7]. Globally, less than one-third (29%) of the children aged 6–23 months met the minimum dietary diversity (MDD), that is, children who ate foods from more than or equal to four (out of seven) groups on the previous day, contributed poor physical growth, including irreversible outcomes of stunting and poor cognitive development [8, 9].

Dietary diversity is influenced by factors associated with socio-demographic characteristics, parental education, the age of mothers [10], maternal occupation, age and birth order of children [11, 12], mothers’ involvement in child food cooking demonstration programs [13, 14] and antenatal care(ANC) service utilization [15, 16] .

Ethiopia has been implementing different interventions to reduce the magnitude of the problem. For example, the Sustainable Undernutrtion Reduction in Ethiopia (SURE) is one of the first government-led multi-sectoral programmes for the improvement of nutrition outcomes that particularly focuses on the integration of the health and agriculture sectors. This program has been implemented in 150 districts on 1.5 million stunted children living in the agrarian districts of Oromia, Amhara, SNNP and Tigray regions with the aim of reducing stunting to 26% by 2020 and improving complementary feeding and dietary diversity. It provided nutrition education using the behavior change communication (BCC) approaches [17].The project has such three main components as enhancing community-based nutrition (CBN) to address inadequate complementary feeding, improving household dietary diversity through IYCF and familiarizing nutrition-sensitive agriculture.

Existing empirical evidence is mainly found in the northern, western, and other parts of Ethiopia. However, only limited studies compared the dietary diversity and associated factors among SURE project covered and uncovered district mothers of children 6–23 month of age in West Gojjam zone [17]. Therefore, this study aimed to compare the proportions of dietary diversity among SURE program covered and uncovered districts and identify factors associated with dietary diversity among young children aged 6–23 months.

## Methods

### Study design and setting

A community- based comparative cross-sectional study was conducted in three selected districts of West Gojjam zone from February 29 to April 20, 2019. West Gojjam is one of the administrative zones in Amhara region, North West Ethiopia. It is located 567 km from Addis Ababa, the capital of Ethiopia, and has 16 districts and 444 kebeles. SURE and Save the children programs which were working to strengthen existing efforts in the country covered four of the 16 districts each, that is eight districts. A total of 117,673 mothers who had young children aged 6 to 23 months lived in the SURE covered (Yilimanadennsa) and the uncovered (Bahir-Dar Zuriya and Debub Achffer districts) which largely dependent on agriculture. The zone had 3 hospitals, 104 health centers, and 391 health posts that providing health services including maternal and child health care.

### Study population and sampling procedure

All infants and young children aged 6–23 months and their mothers who had lived for at least 6 months in the area participated in the study. As this was a comparative cross-sectional study, the minimum sample size was determined by using the double population proportion formula with the assumptions exposed (intervention applied) and unexposed (intervention not applied) groups. To estimate the minimum sample size, a dietary diversity proportion (13%) was taken as p2 from a previous study [18]. However, since there has been no previous finding for the intervention group, the assumption that intervention increases the proportion of dietary diversity by 15% p1 yielded 28%. The final sample size was calculated using the Epi Info software with the assumption of a 95% confidence interval, 80% power, 1:1 ratio of exposed to unexposed, 3% design effect, and 10% non-response rate. Therefore, the final minimum adequate sample size was 832. A multistage stratified sampling and the simple random sampling technique was employed to select study participants in West Gojjam zone. Initially, districts were categorized as SURE program covered and uncovered. Three districts, one covered and two uncovered were selected using the lottery method for the study. The three selected districts had a total of 85 kebeles (35 covered and 50 uncovered).Out of the 85kebeles, seven in SURE covered and ten in uncovered districts were selected using the lottery method. Participants were proportionally assigned to each kebele using the community-based demographic and health related information registration book of health extension workers. Finally, mother to child pairs were selected from each keble using the simple random sampling methods after giving codes to each household which had young children aged 6 to 23 months. If there were more than one children in the households, we selected the index child by the lottery method.

### Operational definitions

#### Adequate dietary diversity

If children (aged 6–23 months) received at least four food groups out of seven in the preceding 24 h of the interview [10, 19].

#### Satisfactory media exposure

Mothers/caregivers of children exposed to media at least once a week by reading newspapers or magazines or listening to the radio or watching TV [11]..

#### Good knowledge

Knowledge of mothers about child feeding, if the mothers answered seven knowledge questions out of the ten they have good knowledge [20].

#### Household food insecurity

HFIAS (household food insecurity access scale) was assessed from FANTA (Food and Nutrition Technical Assistance) 2007 with nine main question, HFIAS divided into (Food security defines the Household food security level of the summations were ≤ 1 point out of 27 scores while the household food security level of the summations ≥2 points out of 27 scores were food insecure) [21].

### Data collection tool and procedure

Data was collected through a face to face interview, using a structured and pre-tested questionnaire.

In order to maintain the quality of data, 2 days training was given to data collectors and supervisors by the principal investigator. A 5% pretest was conducted in non selected districts, and the questionnaire was initially prepared in English and translated to Amharic and retranslated to English by language and public health experts to guarantee consistency. On-site supervision was performed, and each copy of the questionnaire was checked for completeness and accuracy before data entry; 17 clinical nurses and six BSc graduate nursing or public health field supervisors were involved in the data collection process.

### Methods of assessment

Dietary diversity practice was collected and calculated as the sum of the number of different food groups consumed by the child in the 24 h prior to the assessment. The list of food groups included, grains, roots, and tubers; legumes and nuts; dairy products (milk, yogurt, cheese); flesh foods (meat, fish, poultry and liver/organ meats); eggs; vitamin-A rich fruits and vegetables and other fruits and vegetables. Finally, if respondents consumed four or more of the food groups, they were considered as having adequate dietary diversity [2].

Household wealth index adopted from EDHS 2011 was determined using the Principal Component Analysis (PCA) by considering household assets, such as livestock, type of house, durable assets and productive assets. First, variables coded between 0 and 1 were entered and analyzed using PCA; then variables with commonality values of greater than 0.5 were used to produce factor scores. Finally, the factor scores were summed and ranked as “poor”, “medium” and “rich”.

Food insecurity was measured using the FANTA (Food and Nutrition Technical Assistance Tool) household food insecurity access scale (HFIAS) [21]. It consisted of nine “occurrence questions” that represented a generally increasing level of severity of food insecurity (access) and nine “frequency-of-occurrence” questions that asked as a follow-up to each occurrence question to determine how often the condition occurred. The frequency-of-occurrence question was skipped if respondents reported when the condition described in the corresponding occurrence question was not experienced in the previous 4 weeks (30 days). Finally, individuals were considered as food secure, if they said “no” to all items or just experienced worry but rarely; mildly food insecure households were those who were defined sometimes or often worried about not having enough food and/or unable to eat favorite foods and/ or rarely ate a more monotonous diet than desired. Households that reported they rarely or sometimes ate more monotonous diets than desired sometimes or often and/or had started to cut back on quantity by reducing the size of meals or the number of meals were coded as moderately food insecure.

### Data processing and analysis

Data were entered into EPI INFO version 7 and analyzed using the Statistical Package for Social Sciences (SPSS) version 20. Descriptive statistics, including frequencies and proportions were used to summarize the variables. A binary logistic regression model was fitted to identify factors associated with dietary diversity practices. Variables with *P*–values of < 0.2 in the bi-variable analysis were entered in to the multivariable analysis to control possible effects of confounders. The Adjusted Odds Ratio (AOR) with a 95% of confidence interval was used to examine the strength of associations, and a *P*– values ≤0.05 was used to declare statistical significance in the multivariable analysis.

## Result

### Socio-demographic and economic characteristics of the respondents

A total of 832 mother-child pair participated in the study with a 100% response rate. Out of these 416 (50%) were from SURE program covered district. Over half, 54.6 and 61.5% of the respondents from SURE covered and uncovered areas, respectively, were in the age range of 25–34 years with the mean (±SD) of 28.75(±5.935) years. 248 (59.5%) of the mothers in the SURE covered and 251(60.3%) of uncovered districts were unable to read and write; 228 (54.8%) children aged 6–23 months in the covered and 259(62.3%) in the uncovered districts were second to fifth birth order. Nearly one third of the respondents in both groups had poor family wealth status (Table 1).

**Table 1:** Socio-demographic and economic characteristics of children and caregivers among SURE covered and none covered districts in West Gojjam Zone, 2019

Socio-demographic	SURE, covered district *n* = 416			Non-SURE covered *n* = 416	
Variable	Category	Frequency	%	Frequency	%
Age of mother	15–24 year	99	23.8	92	22.1
	25–34 year	227	54.6	256	61.5
	35–50 year	90	21.6	68	16.3
Marital status of the mother	Single	44	10.6	9	2.2
	Married	355	85.3	394	94.7
	Divo/widow/separated	17	4.1	13	3.1
Religion	Orthodox	384	92.3	405	97.4
	Muslim	23	5.5	11	2.6
	Protestant	8	1.9		
Educational status of the mother	unable to read and write	202	48.6	248	59.6
	able to read and write	143	34.4	102	24.5
	Primary	31	7.5	38	9.1
	Secondary	29	7.0	23	5.5
	Certificate and above	11	2.6	5	1.2
Maternal Occupation	Farmer	281	67.5	251	60.3
	Government worker	44	10.6	46	11.1
	House wife	91	21.9	119	28.6
Educational status of the father	unable to read and write	121	29.1	29.1	29.1
	able to read and write	185	44.5	44.5	73.6
	Primary	55	13.2	13.2	86.8
	Secondary	27	6.5	6.5	93.3
	certificate and above	28	6.7	29.1	29.1
Father’s Occupation	Farmer	331	79.6	350	84.1
	government worker	22	5.3	6	1.4
	Merchant	17	4.1	4	1.0
	other^a^	46	11.1	55	13.2
Relation of respondent	grand moth			3	.7
	Mother	416	100.0	413	99.3
Age of the child	6–11 month	213	51.2	177	42.5
	12–17 month	117	28.1	142	34.1
	18–23 month	86	20.7	97	23.3
Birth order	first order	164	39.4	118	28.4
	second to fifth	228	54.8	259	62.3
	above fifth	24	5.8	39	9.4
Family size	< 4	197	47.4	156	37.5
	**> = 4**	219	52.6	260	62.5
wealth status	Poor	135	32.5	137	32.9
	Medium	142	34.1	140	33.9
	Rich	139	33.4	138	33.2

^a^= head of priests, guard

### Maternal and child health care related factors of the study participants

Almost all of the participants (92% of SURE covered and 97% of uncovered) had at least one ANC service, while 263(63.2%) of mothers under SURE and 243(58.4%) out of SURE had PNC services; 348 (83.7%) children under and 282 (67.8%) out of SURE took Vitamin A supplementations (Tables 2 and 3).

**Table 2:** Maternal and child health-related factors of the study participants in west Gojjam Zone, North west Ethiopia, 2019

Variable	Category	SURE, covered district *n* = 416		None covered district *n* = 416	
Variable	Response	Frequency	%	Frequency	%
Place of delivery	Hospital	119	28.6	56	13.5
	H/C and H/P	262	63.0	310	74.5
	HOME	35	8.4	50	12.0
Time reaches H/F	less than two hours	403	96.9	402	96.6
	2 h and above	13	3.1	14	3.4
ANC follow up on H/F	Yes	385	92.5	405	97.4
	No	31	7.5	11	2.6
PNC follow up	Yes	263	63.2	243	58.4
	No	152	36.5	172	41.3
Vaccine	No	0	0	4	1.0
	Yes	416	100.0	412	99.0
GMP	No	113	27.2	270	64.9
	Yes	303	72.8	146	29.8
Vitamin A supplementation	No	68	16.3	134	35.1
	Yes	348	83.7	282	67.8

**Table 3:** Source of Nutrition information about dietary diversity and child feeding

Variable	Category	SURE, covered district		None covered district	
Variable	Response	Frequency	%	Frequency	%
Heard information about child feeding	Yes	384	92.3	382	91.8
	No	32	7.7	34	8.2
Exposure media	Satisfactory	139	33.4	67	16.1
	Un satisfactory	277	66.6	349	83.9
Nutrition counseling during ANC	Yes	370	88.7	380	91.3
	No	46	11.1	36	8.7
nutritional counseling during PNC	Yes	309	74.3	184	44.2
	No	107	25.7	232	55.4
IYCF counseling	Yes	194	46.6	72	17.3
	No	222	53.4	344	82.7
HEW/AEW food demonstration	Yes	207	49.8	80	19.2
	No	209	50.2	335	80.5
HEW& AEW house visit	Yes	189	45.4	28	6.7
	No	226	54.3	388	93.3

### Mother’s on dietary diversity and child feeding knowledge

**Fig. 1:**
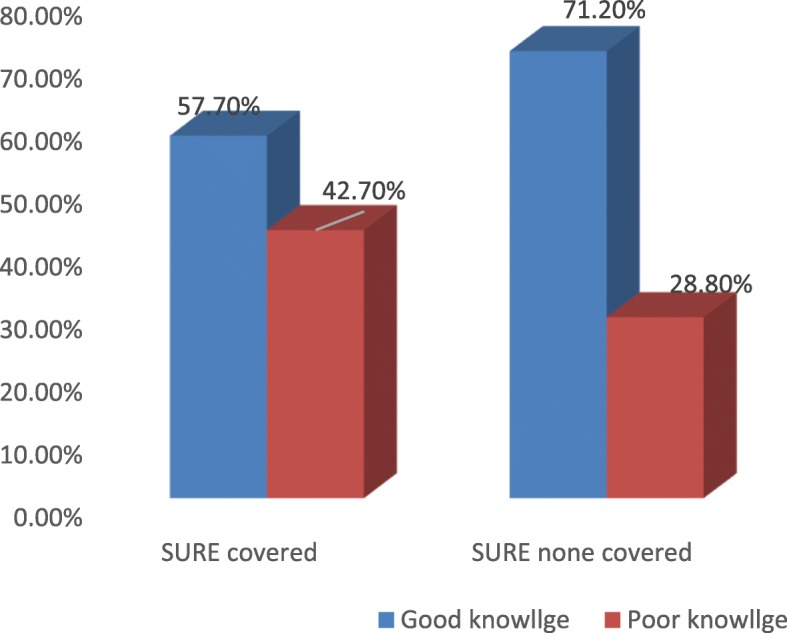
Knowledge of mothers on dietary diversity and child feeding in west Gojjam zone, 2019

Two hundred forty (57.57%) of SURE covered and 296(71.2%) of non covered caregivers had good knowledge about dietary diversity and child feeding (Fig. 1).

### Household food insecurity status and information about nutrition

**Fig. 2:**
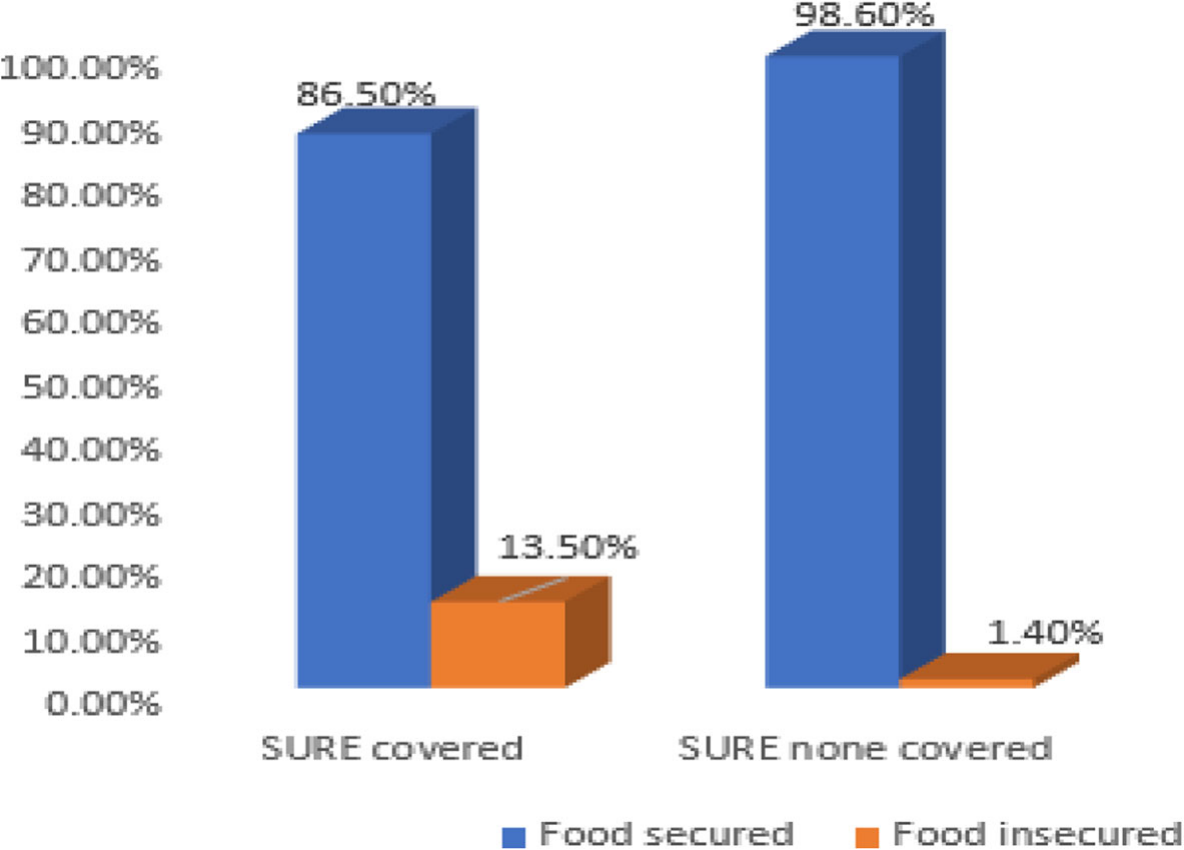
Household Food Insecurity Access Scale (HFIAS) in west Gojjam zone, 2019

Of the mothers,92.3% in SURE covered and 91.8% in the uncovered districts had information about child feeding; 88.3% of the mothers in the covered and 91.7% in the uncovered districts obtained the information during their ANC follow ups,while 207(49.8%) and 80(19.2%) in the covered and uncovered districts, respectively, gathered information from HEW/AEW at food demonstration programs. In the covered and uncovered districts, 360(60.5%) and 410(98.6%) of the households were food secure respectively (Fig. 2).

### Dietary diversity

**Fig. 3:**
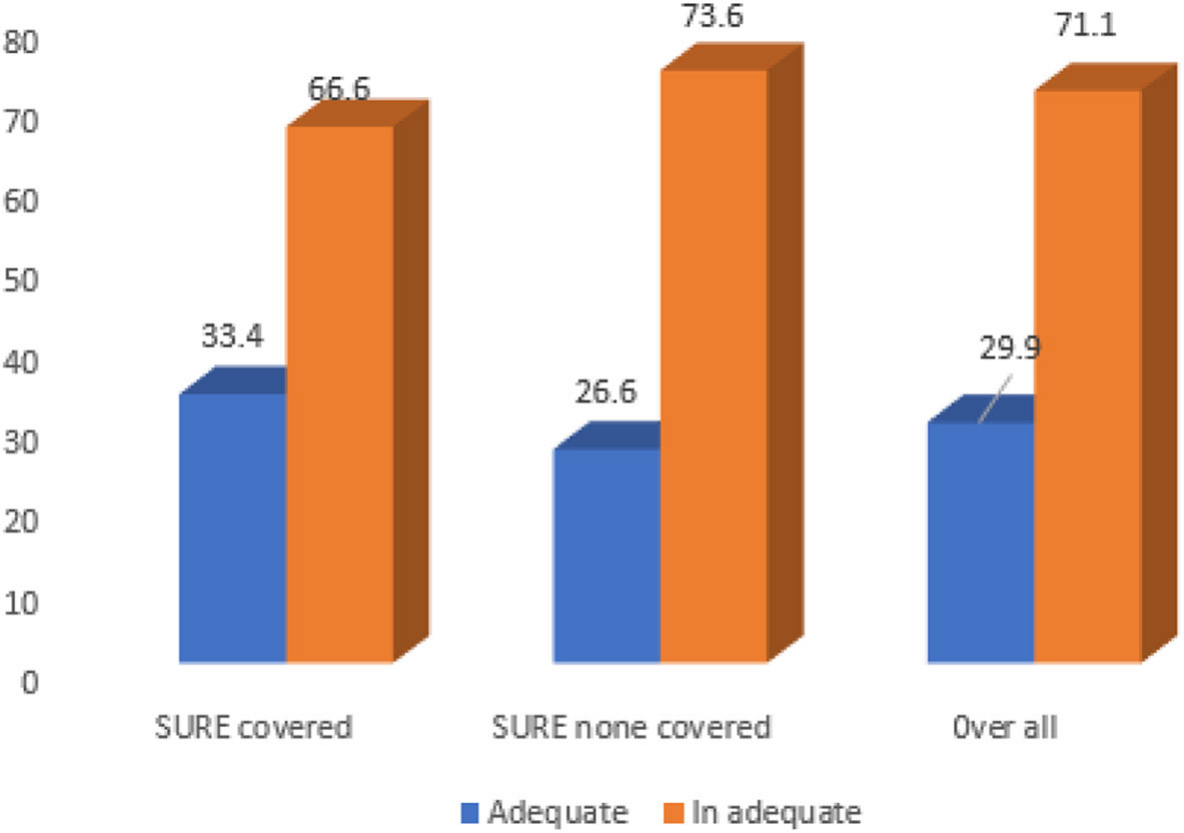
Dietary diversity score among children aged 6–23 months in SURE covered and uncovered district in west Gojjam zone, Northwest, Ethiopia, 2019

The overall proportion of children who received adequate dietary diversity in the study area was 29.9% at (95% CI: 27–33%). The Pearson chi2-square test illustrated that there was a statistically significant adequate dietary diversity difference in the SURE program covered and uncovered districts (pr = 0.028). Lower proportion of adequate dietary diversity was noted among children in the uncovered districts 26.4% (95%CI: 22–31%) than covered district which was 33.4% (95%CI: 29–38%) (Fig. 3).

### Factors associated with adequate dietary diversity in West Gojjam zone

The result of the multivariate analysis revealed that SURE coverage of districts, ANC visits, PNC follows ups, GMP, Vit A supplementation, child food demonstration, and HEW/ AEW household visits were found to be statistically and independently associated with adequate dietary diversity in the study area.

Children who were covered by SURE were 2.5times [AOR = 2.55,95%CI:1.63, 3.97] more likely to have adequate dietary diversity compared to those who were not covered, and children who had GMP were 2.7 times [AOR = 2.74,95%CI:1.80, 4.18] more likely to take diversified diets than those who had not. Children whose mothers had PNC follow ups during their last childbirth were 2.1times [AOR = 2.13, 95%CI: 1.38, 3.28] more likely to have adequate dietary diversity than their counterparts. Also, children who received vitamin A supplementation were almost 2 times [AOR = 2.10, 95%CI: 1.22, 3.61] more likely to have diversified diets than those who didn’t receive. Children whose mothers had received food demonstration trainings were 1.9 times [AOR = 1.88, 95% CI: 1.19, 2.96] more likely to have adequate dietary diversity compared to those whose mothers didn’t receive trainings. Mothers whose houses were visited by HE/AEWs were 2 times [AOR = 2.00, 95%CI: 1.25, 3.21] more likely to have adequate dietary diversity than those whose mothers’ houses were not visited (Table 4).

**Table 4:** Factors associated dietary diversity among children aged 6–23 months in SURE covered and non- covered and districts in West Gojjam zone

Variable	Category	Dietary Diversity		COR (95% CI)	AOR (95% CI)
		Adequate	Inadequate		
SURE covered	Yes	139	277	1.39(1.04, 1.88)	2.55(1.63, 3.97) **
	No	110	306	1	1
ANC visit	Yes	190	377	1.76(1.26, 2.47)	1.72(1,16, 2.55) **
	No	59	206	1	1
PNC follow	Yes	208	298	4.96(3.41,7.21)	2.13(1.38,3.28) **
	No	40	284	1	1
GMP	Yes	191	258	4,15(2.96,5.81)	2.74(1.80,4.18) **
	No	58	325	1	1
Vitamin Supplementation	Yes	227	407	4.46(2.78,7.15)	2.10(1.22,3.61) **
	No	22	176	1	1
Nutrition information	Yes	240	509	2.89(1.41, 5.33)	1.47(0.64, 3.38)
	No	9	73	1	1
Exposure media	Satisfactory	117	89	2.23(1.53,3.07)	1.38(0.92,2.06)
	Un satisfactory	461	165	1	1
counseling ANC	Yes	240	509	3.82(1.88, 7.77)	1.52(0.66, 3.46)
	No	9	73	1	1
counseling PNC	Yes	194	299	3.35(2.38,4.77)	1.36(0.88, 2.12)
	No	55	284	1	1
IYCF counseling	Yes	137	129	4.35(2.974,5.584)	1.59(0.99,2.06)
	No	117	454	1	1
Food demonstration	Yes	143	144	4.103(2.99,5.61)	1.88(1.19,2.96) **
	No	106	438	1	1
HEW house visit	Yes	111	106	3.63(2.63,5.06)	2.00(1.25,3.21) **
	No	137	477	1	1

**= Statistically significant at *p* < 0.001

## Discussion

The overall proportion of adequate dietary diversity among children 6 to 23 months of age in this study was 29.9% (95% CI: 27–33%), while that of adequate dietary diversity among children in SURE covered and uncovered districts was 33.4% (95%CI: 29–38%) and 26.4%(95%CI: 22–31%), respectively. The chi-square test showed that there was a statistically significant difference in dietary diversity between SURE covered and uncovered districts (*p*-value =0.028).

The proportion of adequate dietary diversity in the study area was 29.9% in line with findings in Nepal (30.4%) [15], Bale zone (28.5%) [16], and Wilayat Sodo, Ethiopia (27.3%) [22].It was lower than reports of studies in Bench Maji zone, Southwest Ethiopia (38%) [23],and Wolaita zone, Southern Ethiopia (41.9%) [24]. The possible reason might be that Ethiopia as a multiethnic country has diverse geographic and weather conditions which may affect the accessibility of adequate dietary diversity. However, our result was higher than those of studies conducted in the Slum areas of Bahir Dar city (20%) [25], Dangilla, northwest Ethiopia (12.6%) [12], Gorche district, Southern Ethiopia(10,6%) [13] and North Wollo zone, north east Ethiopia (7%) [26]. The possible reason might be differences in study times or times of data collection. It might also be due to the fact that North Wollo zone was repeatedly affected by food insecurity compared to the current study area.

Though the proportion of adequate dietary diversity noted in this study looks better than what was reported by the Ethiopia Demographic and Health Survey (EDHS 2016), (14%). The rate of adequate dietary diversity was by far lower than the national target (40%) stated in the National Nutrition Program (NNP2020). This difference might be due to the fact that SURE program is a government-led multi-sectoral intervention that aims to integrate the work of the health and agriculture sectors to improve child feeding through behavior change communication [17].This behavior change intervention also provides an opportunity for complementary feeding, nutrition counseling to mother to improve the intake of adequate dietary diversity by enhancing the existing Community-Based Nutrition (CBN) programs.

The proportion of adequate dietary diversity among children who lived in SURE covered districts was 33.4%. This finding is in line with that of a study conducted in Nepal (30.4%0 [15] and Bench Maji zone, southwest Ethiopia, (38%) [23]. This might be due to similar socio-demographic and economic characteristics of the communities. Our finding was lower than those of studies conducted in Sri Lanka (71%) [27], Indonesia (58.2%) [28] and Ghana(47.7%) [29]. The possible explanation might be socio-demographic characteristics and cultural differences which may affect the dietary diversity of children. In this study, the rural areas have culturally almost homogenous population with similar feeding practices, and nearly half (48.6%) of the mothers were unable to read and write, however. In Ghana, only 28.4% of the caregivers were unable to read and write. Therefore, uneducated mothers might not easily understand the consequences of undiversified diets and the nutritional requirements of infants and young children. Studies in Uganda also showed that caregivers who participated in a 10-week nutrition education program improved their dietary diversity score by 3 to 10.3%, indicating that mother’s educational status had its own effect on the dietary diversity of children [14]. In addition, the proportion of DDS in this study was lower than a finding in Addis Ababa(59.9%) [20]. This difference might be due to the fact that Addis Ababa is the capital of Ethiopia, where subjects might have better exposure to the media and health services related to dietary diversity and child feeding practices. The finding is supported by a study in the northern part of Ethiopia [12] and the secondary analysis of the Ethiopian health survey [10].

The proportion of dietary diversity among children aged 6–23 months under the program was also higher than those of EDHS 2016(14%),the systemic review in Ethiopia(23.25%) [18], Sinana district, Ethiopia (13%) [18],Dabat district (17%) [11],and Kemba district (23.3%) [30]. The difference might be that the study area (Yilimana Denssa district) was under SURE program where HEW/AEW gave supportive training and counseling to mothers regarding the preparation of complementary foods [17].

SURE covered districts were 2.5 times more likely to have adequate dietary diversity than the uncovered once. This was similar to the result of a study conducted in Basona Worana distirct, Amhara region [17].The Possible reason might be that SURE program mainly integrated health& agriculture through the Community Based Nutrition Service, Enhanced Nutrition Service, Systems Strengthening and multisectoral coordination to enhances the diversity of diets.

Vitamin A supplementation had a statistically significant association with dietary diversity. Mothers who had child health nutrition services (Vitamin A supplementation) had better dietary diversity than those who had no child health nutrition services. The possible reasons might be that West Gojjam zone of all the districts was supported by the Micronutrient Initiative (MI), which focused on strengthening and integrating delivery platforms for micronutrients interventions by increasing the number of children receiving vitamin A supplementation starting at the 6-month. Therefore, this service increased mother’s health-seeking behavior and routine communication with HEWs and improved awareness about adequate dietary diversity in child feeding [31–33].

Mothers who had ANC (Antenatal care) visits during pregnancy were more likely to provide the recommended dietary diversity for their children compared with their counterparts. This finding, supported by a study in north west Ethiopia [12, 29] .This could be due to the fact that antenatal care services improve maternal counseling and community conversation programs on child feeding practices enhance the understanding of mothers on how to prepare and feed children.

Mothers who had postnatal care visits were more likely to provide the recommended dietary diversity compared to their counterparts. This finding was supported by findings in South Asian countries [25, 34]. The possible reasons for the association might be counseling about complementary feeding practice and demonstrations of diversified complementary food during post natal periods.

For mothers whose houses were visited by H/AEWs the odds of adequate dietary diversity were two times better than those of their counterparts because SURE provides trainings which contribute to improved IYCF and nutrition-sensitive agricultural knowledge for HEWs/AEWs. One of the components of SURE focuses on household visits which provide exposes both mothers and fathers to key messages. Similar findings were reported by the SURE evaluation study [17] and the global nutrition target 2025 [35].

Growth Monitoring and Promotion were found to be significantly associated with adequate dietary diversity. Children who had got Growth Monitoring and Promotion services were 2.74 times more likely took diversified than those who didn’t have GMP services. This finding is similar to those of studies in Burkina Faso [35],northwest Ethiopia [11, 36],and southern Ethiopia [30].Mothers involved in monthly growth monitoring follow-ups were more likely to provide the recommended dietary diversity and meal frequency and enhance their understanding of how to prepare and feed children with diversified foods than those who were not involved.

Finally, mothers who took food demonstration practices were 1.88 times more likely to provide adequate dietary diversity than their counterparts. This was supported by a study in Gorche district, southern Ethiopia [13].From the demonstration mothers acquire skills and confidence to select food mixtures and prepare improved child and family feeding recipes using locally available and affordable foods and provide systems for peer learning through the promotion of nutritionally-sound indigenous knowledge. In addition, cooking demonstrations enhance skills, strengthen extension workers’ capacity to promote and facilitate dietary diversification and the adoption of improved family feeding practices by communities [37–39].

### Strengths and limitations

That we compared the impact of SURE by using the program covered districts as intervention and the uncovered ones as control through a comparative study design is perhaps the strength of the work. On the other hand, the fact that the measurements of dietary diversity were prone to recall bias might be the weakness although data collectors did their best to probe mothers to recall the food items they gave to their index child. Besides, the study could not claim to be free from social desirability bias in the measurement of dietary information.

## Conclusion

This study revealed that the proportion of dietary diversity among children aged 6–23 months in SURE covered districts was higher than that of children living in the uncovered districts. ANC and postnatal care services, participation in food preparation programs and household visits by health extension workers were significantly associated with dietary diversity. Therefore, scaling up and strengthening the program to uncovered districts and providing health and nutrition counseling on Infant and Young Child Feeding (IYCF) during maternal ANC and PNC services are recommended for achieving the recommended dietary diversity.

## Data Availability

The datasets used and/or analyzed during the current study are available from the corresponding author on reasonable request.

## Abbreviations

AEW: Agricultural Extension Worker
ANC: Antenatal Care
CBN: Community-Based Nutrition
CI: Confidence Interval
COR: Crude odds ratio
DD: Dietary Diversity
EDHS: Ethiopia Demography Health Survey
GMP: Growth Monitoring and Promotion
HEW: Health Extension Worker
HFIAS: Household Food Insecurity Access Scale
HHs: Households
IYCF: Infant Young Child Feeding
MDD: Minimum Dietary Diversity
PNC: Postnatal Care
SNNPR: South Nation and Nationalities People Region
SPSS: Statistical Package for Social Science
SRS: Simple Random Sampling
SURE: Sustainable Under nutrition Reduction in Ethiopia
WHO: World Health Organization
